# Mucolytic and Antioxidant Properties of Carbocysteine as a Strategy in COVID-19 Therapy

**DOI:** 10.3390/life12111824

**Published:** 2022-11-08

**Authors:** Andrea Bianco, Stefano Conte, Domenica Francesca Mariniello, Valentino Allocca, Maria Gabriella Matera, Vito D’Agnano, Luigi Lanata, Mario Cazzola, Fabio Perrotta

**Affiliations:** 1 Department of Translational Medical Sciences, University of Campania “L. Vanvitelli”, 80131 Naples, Italy; 2 U.O.C. Clinica Pneumologica “L. Vanvitelli”, A.O. dei Colli, Ospedale Monaldi, 80131 Naples, Italy; 3 Unit of Pharmacology, Department of Experimental Medicine, University of Campania “L. Vanvitelli”, 80131 Naples, Italy; 4 Medical Department, Dompé Farmaceutici SpA, 20122 Milan, Italy; 5 Department of Experimental Medicine, University of Rome Tor Vergata, 00133 Rome, Italy

**Keywords:** mucolytics, Carbocysteine, COVID-19, SARS-CoV-2, COPD, antioxidants

## Abstract

SARS-CoV-2 infection leads to a heterogenous spectrum of clinical conditions ranging from self-limiting upper airway infection to severe respiratory failure. Carbocysteine is a thioether mucolytic with antioxidant and anti-inflammatory activities. Carbocysteine has been shown to have anti-viral effects on human rhinovirus, RSV and the influenza virus as well as interfering with upper airway ciliary motility, the first site of SARS-CoV-2 infection, leading to more effective mucus clearance and potential containment of viral spread towards the lower airway. Positive effects, in terms of limiting superimposed bacterial infection and reducing oxidative stress, have also been documented in COPD patients. Accordingly, Carbocysteine should also be considered in both post-exposure prophylaxis and early-phase treatment of COVID-19 in combination with other agents (monoclonal antibodies, antivirals, non-steroidal anti-inflammatory agents, and inhaled corticosteroids). In this review, we explored the pharmacokinetic and pharmacodynamic aspects of Carbocysteine to delineate its potential therapeutic impact in patients with COVID-19.

## 1. Introduction

On the 11th of March, 2020, the WHO declared the 2019 novel coronavirus disease (COVID-19) a pandemic infection, caused by a novel beta-coronavirus known as Severe Acute Respiratory Syndrome Coronavirus 2 (SARS-CoV-2). Experimental evidence has confirmed that ACE2 (Angiotensin-converting enzyme 2) is utilized by SARS-CoV-2 as the main entry point into cells. SARS-CoV-2 spike protein consists of two functional regions named the S1 unit—responsible for binding with the host cell receptor ACE2—and S2 [1]. The spike protein can be cleaved by the protease TMPRSS2 (Transmembrane Serine Protease 2) between the S1/S2 units, leading to fusion with the cellular membrane mediated by the S2 unit. Alternatively, after binding with ACE2, SARS-CoV-2 can use the endocytic pathway [2]. Subsequently, SARS-CoV-2 intracellular replication, along with a complex interplay with the immune system, may induce, in a minority of patients, a “Cytokine storm” which has been closely linked with mortality in COVID-19 patients [3]. SARS-CoV-2 is also able to stimulate the production of free reactive oxygen species (ROS) which include various chemical forms (superoxide, peroxides, hydroxyl radical, singlet oxygen and alpha oxygen). ROS cause oxidative damage to various cellular components such as DNA, RNA, lipids and proteins. Besides causing tissue injury, ROS can also amplify inflammation through upregulation of expression of multiple genes, such as IL-6, TNF-α, and chemokines [4,5]. COVID-19 disease can range from mild forms with fever, dyspnea and cough, to severe forms, especially in the elderly with comorbidities, characterized by pneumonia and severe acute respiratory distress syndrome (ARDS) [6,7]. In critical disease forms, rapidly progressive multiple organ failure occurs [8]. These complications seem to be related to the cytokine storm, in which viral load triggers an uncontrolled release of cytokines and abnormal immune response [9]. Moreover, oxidative stress imbalance plays an important role in the pathogenesis of various viral infections, including SARS-CoV-2 infection [10]. The maintenance of the disulfide–thiol equilibrium is an important aspect of viral entry and fusion, and can be affected by oxidative stress [11]. Older age, male sex, smoking and comorbidities such as diabetes, obesity, cardiovascular and pulmonary diseases, including chronic obstructive pulmonary disease (COPD), are known risk factors related to severe COVID-19 disease, and all these conditions cause excessive oxidative stress [12,13]. Carbocysteine, a mucoregulatory drug, through its antioxidative and anti-inflammatory effects, could be an ideal candidate for COVID-19 treatment. In addition, Carbocysteine may improve mucociliary clearance and prevent viral protein binding on the host cells in the early disease stages.

On the basis of the established role of mucoregulators in respiratory disorders this review addresses the potential efficacy of Carbocysteine as a form of supportive care in SARS-CoV2 infection.

## 2. Carbocysteine: Pharmacology

**Figure 1:**
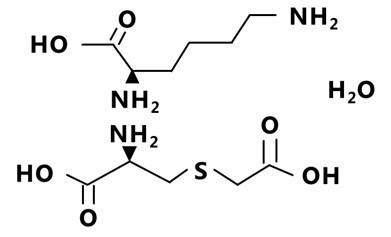
Carbocysteine lysine salt: structural formula.

Carbocysteine (S-carboxymethylcysteine or S-CMC) is a mucoactive drug with antioxidant and anti-inflammatory properties, which is a thioether derivative of amino-acid L-cysteine. Carbocysteine is well-absorbed when taken orally. Oral preparations of Carbocysteine, both as S-CMC and its lysine salt (S-CMC-Lys) (Figure 1), are available. The lysine group is cleaved on gastric absorption to form the active drug S-CMC [14]. The highest serum levels are achieved at 1 to 1.7 h and the plasma half-life is 1.33 h [2]. Local activity is related to high concentrations in lung tissue and respiratory mucus. Urinary excretion of the unchanged form of the drug occurs in approximately 30% to 60% of patients [15]. Metabolic pathways include acetylation, decarboxylation and sulfoxidation to produce inactive derivatives. Significant variability exists in metabolism due to genetic polymorphism in the sulfoxidation capacity. Two cytosolic enzymes are responsible for the metabolism of Carbocysteine: cysteine dioxygenase and phenylalanine 4-hydroxylase [16]. Reduced metabolism can cause increased exposure to Carbocysteine, explaining variable clinical response between patients who may have polymorphisms affecting the enzymes responsible for Carbocysteine metabolism. As for the other mucolytic drugs, due to interference with gastric mucus homeostasis, a hypothetical gastric erosion risk has been reported and specific contraindication for patients with active gastric ulceration has been defined. Clinical studies in humans and animal models did not identify significant drug interaction in protracted use [17].

Healthy mucus is a gel composed of 97% water and 3% solids (mucins, non-mucin proteins, salts, lipids, and cellular debris) with low viscosity and elasticity that is easily transported by ciliary action. Dysregulation in mucus clearance is mainly caused from both exalted mucin deposition and surface liquid volume diminution, leading to a more viscous and elastic mucus. Additionally, these changes cause a raised adherence of the mucus to the airway wall [18,19,20,21,22,23]. Feedback interactions between mucus concentrations and cilia beating, via purinergic signaling, coordinate Na+ absorptive vs. Cl- secretory rates to maintain mucus hydration in health. In several respiratory diseases, mucus dehydration occurs. Multiple mechanisms derange the ion transport pathways that normally hydrate mucus in lung diseases, such as cystic fibrosis (CF), chronic obstructive pulmonary disease (COPD), bronchiectasis, and primary ciliary dyskinesia (PCD). A key step in muco-obstructive disease pathogenesis is the compression of the mucus layer into the airway surface with the formation of adherent mucus plaques and plugs, particularly in distal airways. Mucus plugs create locally hypoxic conditions and produce airflow obstruction, inflammation, infection, and, ultimately, airway wall damage [10].

Fucose and sialic acid contents on sugar chains on mucins are responsible for the viscous properties of the mucus, and increased viscosity has been reported to co-exist with impaired mucociliary transport, vulnerability to bacterial/viral infection and inflammatory cell infiltration in the airway [24].

Despite its modest direct mucolytic activity, Carbocysteine is a mucoregulator drug regulating fucose and sialic acid contents in mucus glycoprotein, likely through the inhibition of the activity changes of fucosidase, sialidase, fucosyltransferase and sialyltransferase, thus reducing mucus viscosity [25]. The major mucins produced in the airways are soluble mucins, such as mucin 5B (MUC5B) and mucin 5AC (MUC5AC), which are secreted by goblet cells. Four days after the start of therapy, a change in the consistency of the sputum had already been documented, which was found to be more fluid. In some studies, it has been shown that Carbocysteine facilitates the entry of amoxicillin into the bronchial secretions of patients suffering from chronic bronchitis. This effect is due to a change in the glycoprotein structure that forms the sputum. Dose regimens for Carbocysteine vary internationally, ranging from 750 mg twice daily to 4.5 g once daily. There is little evidence to advocate one particular dosing regimen, although nocturnal administration may achieve higher concentrations of the active compound [26,27,28].

## 3. Carbocysteine in Pulmonary Diseases

### 3.1. Carbocysteine and COPD

Chronic obstructive pulmonary disease (COPD) is a chronic airway disease, mostly associated with cigarette smoke (CS), characterized by persistent respiratory symptoms, mucus hypersecretion and airflow limitation. COPD is characterized by airway abnormalities, including smooth muscle hypertrophy, mucus accumulation, wall thickening with neutrophil and inflammatory infiltrate, increase in number and size of goblet cells and pulmonary emphysema as a consequence of loss of alveolar attachments [29]. Oxidative stress may play an important role in COPD pathogenesis. In fact, oxidative stress biomarkers are increased in exhaled breath condensate, sputum, and blood samples of COPD patients. Oxidative stress drives COPD through the activation of several mechanisms, including the proinflammatory transcription factor nuclear factor-KB (NF-κB) [30], the p38 mitogen-activated protein kinase (MAPK), the generation of autoantibodies to carbonylated proteins, the reduced expression of sirtuin-1 and histone deacetylase HDAC2, DNA damage, the reduced activity of antiproteases and the increased release of transforming growth factor (TGF)-β, as well as lipid peroxidation products such as malondialdehyde (MDA). Over 50 cytokines and chemokines are released in the lungs of patients with COPD. Oxidative stress represents a treatable target in patients with COPD. This could be achieved either by decreasing the generation of oxidants or by enhancing antioxidant activities. Antioxidant agents, such as thiol compounds/donors and their analogs (GSH and mucolytic drugs, such as N-acetyl-L-cysteine, Carbocysteine, and erdosteine), scavenge free radicals/oxidants, increase intracellular thiol levels and control NFκB activation inhibiting inflammatory gene expression. These antioxidant molecules have been shown to improve symptoms and reduce the decline in lung function and the frequency of exacerbations [31], with favorable impact on quality of life [32].

In COPD in vitro models of bronchial epithelial cells exposed to cigarette smoke extracts (CSE), Carbocysteine reduces oxidative stress, increases HDAC2 and HDAC3 expression inducing deacetylation processes, reduces pro-inflammatory markers (IL-8 mRNA, IL-8 release, Toll-like receptor-4 (TLR4) expression, lipopolysaccharide (LPS) binding) and inhibits neutrophil chemotaxis [33,34,35]. Additionally, in vivo studies carried out in COPD patients confirm that Carbocysteine downregulates parameters of systemic inflammation and oxidation, increasing circulating soluble receptor for advanced glycation end products (sRAGE), reducing serum levels of microRNA-21 (miR-21), IL-8 and fluorescent advanced glycation end products (fAGEs), and decreasing concentrations of 8-isoprostane and IL-6 in exhaled breath condensate [36]. Neutrophil influx into the airways has been reported in patients with COPD [37], and human neutrophil elastase (HNE) produces ROS and induces MUC5AC mucin secretion and goblet cell hyperplasia. Carbocysteine reduces HNE-induced mRNA expression and protein secretion of MUC5AC. Carbocysteine also reduces ROS production in the cells induced by HNE. Reduction of HNE-induced mucus secretion by Carbocysteine in the pulmonary epithelial cells may partly relate to the reduction of ROS [38]. Acute worsening in respiratory symptoms requiring adjustment in stable therapy—acute exacerbations (AE)—may occur in COPD patients induced by viral or bacterial infections, exposure to gases, pollution or hemodynamic causes. Acute exacerbated COPD is associated with accelerated lung function loss both in current and former smokers and limits the tolerance of physical exercise and the quality of life, resulting in increased hospitalization rates and overall mortality for both respiratory and non-respiratory causes [39,40]. Previous studies in large COPD populations documented that AEs increase the relative risk for myocardial infarction and stroke [41]. The systemic spillover of pro-inflammatory mediators (IL-6, IL-8, tumor necrosis factor α) from the lungs, prompting platelet activation, endothelial inflammation and atherosclerosis has been considered the major pathogenic mechanism of exalted cardiovascular risk after acute exacerbated COPD [42,43,44]. Randomized clinical trials have shown that daily administration of Carbocysteine for prolonged periods (for 6 or 12 months) reduce COPD exacerbations independently of broncodilatator therapy use and improve lung function and quality of life. A possible explanation could be that Carbocysteine modulates the expression of ICAM1a, which is a receptor for the rhinovirus pathogens most commonly implicated in the genesis of the common cold [45,46,47,48,49,50]. Adhesion of pathogens on the surface of airway epithelial cells is essential for respiratory tract infections to occur. Carbocysteine is able to reduce respiratory infection in COPD patients by inhibiting the adhesions of pathogens to the surface of these cells. Streptococcus pneumoniae can cause various invasive and noninvasive infections and most of these infections are preceded by colonization of the pharynx by the bacteria. Carbocysteine inhibited the attachment of S. pneumoniae to human pharyngeal epithelial cells in vitro [49]. Intercellular adhesion molecule-1 (ICAM-1) is required for the entry of a virus into cellular endosomes [45]. Indeed, ICAM-1 is the major cell entry receptor for human rhinoviruses, which in turn are the major trigger for asthma exacerbations as well as COPD exacerbations [46,47,48]. In COPD patients, a high concentration of circulating ICAM-1 has been detected, which is able to recall neutrophils in the lungs, and also leads to an increased risk of viral infections of the airways [50]. The levels of ICAM-1 are down-regulated by Carbocysteine, reducing the incidence of colds and exacerbations in COPD patients [31].

### 3.2. Carbocysteine and Asthma

Very little is known about Carbocysteine use in asthma. Asthma is a chronic inflammatory disease of the respiratory tract characterized by an abnormal increase in eosinophils in the airways, bronchial hypersecretion, and remodeling of the airways [51]. These changes are caused by inflammatory cytokines such as TGF-β1. Some studies have highlighted the ability of Carbocysteine to reduce the expression of TGF-β1 in lung tissues, blocking the remodeling of the airways in asthmatic mice [52,53]. Moreover, chronic nonproductive cough is a main symptom in bronchial asthma. Ishiura Y et al. shows that 4 weeks’ treatment with a mucoregulator drug, Carbocysteine, increased the cough threshold to inhaled capsaicin, the active ingredient of red pepper used as cough-receptor stimulant, in asthmatic patients with restoration of depressed neural endopeptidase (NEP) activity in tracheal tissue. In contrast, another mucoregulator drug, ambroxol hydrochloride, did not affect the cough threshold. Oral administration of Carbocysteine may be a novel therapeutic option in patients with bronchial asthma, especially with cough variant asthma [54]. Although the cough is an important mechanism for the protection and clearance of the airways, chronic cough can profoundly and adversely affect the quality of patients’ lives.

## 4. Carbocysteine and COVID-19

### 4.1. Effects of Carbocysteine on Nasal Mucociliary Clearance

Mucociliary clearance is a form of airway protection against pathogens. The efficacy of mucociliary clearance depends on many factors (ambient temperature, humidity, partial pressure of O_2_, pH, trauma, sulfur dioxide, formaldehyde, ozone, chlorine, smoking, chronic sinusitis, chronic and allergic rhinitis, adenoid hypertrophy, fibrosis cystic, chronic bronchitis, septal deviation, surgery and diabetes), which can reduce its defense capacity. Upper airway epithelium represents the first major site of SARS-CoV-2 infection. Before eliciting an adaptive immune response, SARS-CoV-2 must overcome several constitutive respiratory defense barriers. The first is the mucus covering the respiratory tract’s luminal surface, which entraps inhaled particles, including infectious agents, and eliminates them by mucociliary clearance. SARS-CoV-2 itself negatively affects mucociliary activity and causes prolongation of virus/host cell interaction [55]. The multiplication of SARS-CoV-2 viruses damages ciliated epithelial cells, which lose their motile cilia and are therefore no longer able to perform adequate mucociliary clearance. The functional defense mechanisms of the nasal respiratory epithelium weaken in the early stage of SARS-CoV-2 infection, enabling progression of infection towards the lower airways. Finding pharmacological agents able to counteract infection at the upper airway level, the first site of infection, has the potential to contain the viral spread, facilitating self-limiting disease and better outcomes.

Ciliary beating activities can be assessed by two parameters: ciliary beat frequency (CBF) and ciliary bend angle (CBA). Several sub-stances such as Ca^2+^, cAMP, and intracellular pH (ipH) have been shown to increase CBF and CBA. Moreover, a decrease in intracellular Cl^−^ concentration (i[Cl^−^]) coupled with cell shrinkage has been demonstrated to enhance CBA. Carbocysteine stimulates an increase in CBA and CBF, with an i[Cl^−^] decrease and elevation in ipH in airway ciliary cells. The molecular mechanisms that regulate this Carbocysteine activity in airway cells are activation of the CO_2_/HCO3^−^ cotransporter and CF transmembrane conductance regulator (CFTR). A previous study showed that ipH elevation increases both CBF and CBA by 10% and the i[Cl^−^] decrease increases CBA by 20% [56]. Carbocysteine has a mucoregulator action, inhibiting induced inflammation of the airways by reducing the secretion of abnormal mucous glycoprotein and ciliary damage. This remission of airway inflammation may be associated with Carbocysteine-induced normalization of intracellular mediator cAMP levels in the airways [57]. According to these observations, Carbocysteine plays an important role in the prevention of SARS-CoV-2 infection, improving mucociliary clearance and therefore reducing the entry of the virus into lower airways.

### 4.2. Carbocysteine Effects in Respiratory Viral Infections

Carbocysteine has shown anti-viral effects on commonly detected viruses such as rhinovirus, respiratory syncytial virus (RSV), and influenza virus. Yasuda et al. demonstrated that Carbocysteine reduces intercellular adhesion molecule-1 (ICAM-1) levels and rhinovirus RNA entry into the endosomes, thus inhibiting rhinovirus infection in human tracheal epithelial cells [39].

Furthermore, Asada et al. showed that Carbocysteine reduces the release of inflammatory cytokines in tracheal epithelial cells infected with human RSV [58].

RSV infection increased the production of IL- 1**β**, IL-6 and IL-8. Carbocysteine reduced the production of IL-1**β**, IL-6 and IL-8 after RSV infection [58]. However, Carbocysteine reduced the baseline production of these cytokines prior to RSV infection [39]. Therefore, these findings suggest that the reduced production of these cytokines in the cells treated with Carbocysteine after RSV infection might be caused by two mechanisms: the inhibitory effects of Carbocysteine on RSV infection and its effects on cytokine production. As mentioned above, a relationship has been reported between RSV infection and the development of exacerbations of COPD [59]. Carbocysteine reduces the frequency of the common cold [31,39], and Yamaya et al. [60] also demonstrated that Carbocysteine might inhibit influenza A virus (FluA) infection, partly through the reduced expression of the receptor sialic acid (SA 2,6Gal) in human tracheal epithelial cells. Carbocysteine also reduced the number of acidic endosomes from which virus RNA enters into the cytoplasm, and might inhibit the next processes of viral replication. Furthermore, this mucolytic agent may also modulate airway inflammation induced by FluA virus infection reducing concentrations of proinflammatory cytokines and a monokine, including IL-1, IL-6, and IL-8 through the inhibition of NF-κB activation. Reduced NF-κB might be partly associated with the reduced expression of SA 2,6Gal by Carbocysteine in human tracheal epithelial cells [41].

### 4.3. Carbocysteine Properties and Possible Interferences with SARS-CoV2 Infection

Thiols, potent antioxidant and anti-inflammatory agents, can act against SARS-CoV-2 infection through several molecular mechanisms that regulate cell metabolism. In particular, they are able to downregulate the transcription factors—mainly NF-kB—that promote downstream proinflammatory activity, resulting in exalted inflammatory cells recruitment and activation [30].

Furthermore, other potential thiol mechanisms counteracting SARS-CoV-2 infection have been postulated. Firstly, thiols may interact with Cys motifs on the SARS-CoV-2 envelope, limiting the interaction with the spike protein and finally resulting in altered virion stability. The structural modification of the viral spike proteins and of the endothelial ACE2 protein is favored by the presence of a redox environment which determines the formation of sulfhydryl groups, hindering the disulfide bonds at the level of the binding domains necessary for their interaction [61]. In addition, thiols may attenuate methylglyoxal-induced protein glycation and glycosylation; reduction in advanced glycation end products (AGE) might inhibit inflammatory mediators release in cells infected with SARS-CoV-2 [62].

**Figure 2:**
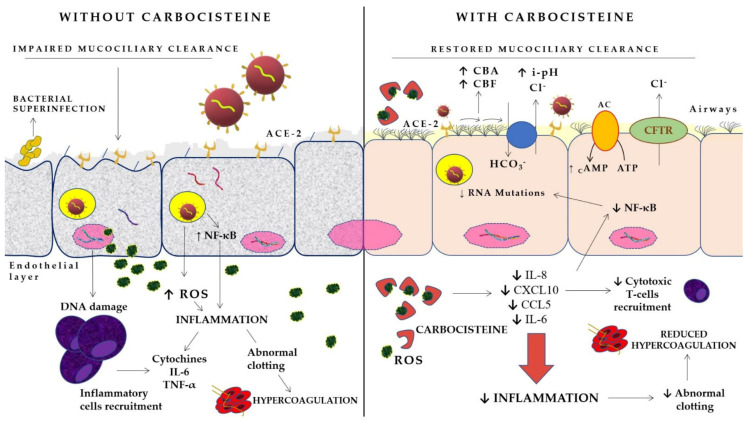
Postulated mechanisms of carbocysteine–SARS-CoV-2–airway cells interaction. AC: adenylyl cyclase; ACE-2: angiotensin converting enzyme-2; CBA: ciliary bend angle; CBF: ciliary beat frequency; CFTR: cystic fibrosis transmembrane conductance regulator; i-pH: intracellular-pH; ROS: reactive oxygen species; TNFα: tumor necrosis factor alpha; ↓: reduced; ↑: increased

Carbocysteine, a thioether, is a powerful scavenger of hydroxyl radicals OH● that could potentially prevent cytokine storms, ROS-induced pulmonary edema and respiratory failure [63,64,65]. RNA viruses need active NF-κB pathway support within host cells in order to replicate. For human coronaviruses, suppression of NF-κB significantly reduces the replication rate. Therefore, drugs that inhibit NF-κB activation could potentially reduce viral replication. This means that, theoretically, Carbocysteine has the potential to inhibit SARS-CoV-2 because of its ability to negatively regulate NF-κB; Carbocysteine inhibited NF-κB activation, including NF-κB p65 phosphorylation and nuclear translocation, and inhibited ERK1/2 MAPK phosphorylation. Carbocysteine reduced the production of IL-6 and IL-8, decreased the expression of inflammatory cytokines such as IL-6 and TNF-α, reduced chemokines such as IL-8, IP-10 and MIP-1β (important factors related to cytokine storms of COVID-19), and exhibited exceptional anti-inflammatory ability [30]. ROS can modify the structure of SARS-CoV-2 through oxidative stress that alters structural and non-structural viral proteins, resulting in the formation of SARS-CoV-2 variants that would allow the pandemic to progress. The cytokine storm causes a dysfunction of the vascular endothelium [66], leading to a state of hypercoagulability and consequently vascular thrombosis [4,5] also favored by the expression of transcription factors activated by oxidative stress and hypoxia [67] (Table 1). Excessive oxidative stress might be responsible for the alveolar damage, thrombosis, and red blood cell (RBC) dysregulation seen in COVID-19 [68]. In effect, free radicals, the downstream product of cytokine storms, are responsible for damage to cells and various organs [69]. In addition, the overproduction of ROS suppresses the T-lymphocyte response, which results in impaired adaptive immunity [70]. These observations suggest a therapeutic value of antioxidants in COVID-19 patients who develop unclear platelet-dependent thrombosis; supplemental Carbocysteine therapy may lead to better cardiovascular outcomes in these patients. Furthermore, Carbocysteine reduces the viscosity of the sputum via modification of the glycoproteins of the bronchial mucus, resulting in mucus elimination; this property of Carbocysteine may help prevent bacterial superinfections that can occur during the course of SARS-CoV-2 (Figure 2).

Multiple clinical studies have reported increased mucus production in severe COVID-19 disease, including increased MUC1 and MUC5AC levels in tracheal aspirates [71,72], repeated airway obstruction due to viscous mucus [73,74], and persistent mucus production during sub-acute to chronic clinical phases [75,76]. Goblet cell metaplasia, characterized by MUC5B-dominated and more variable MUC5AC mucin expression, was observed in COVID-19 tracheobronchial samples. MUC5B was the dominant mucin upregulated in bronchiolar epithelia. Post-SARS-CoV-2-infection-induced inflammatory cell-signaling pathways, including epidermal growth factor receptor (EGFR), and cytokines (IL-1α/β, IL-6, NF-κB) likely mediate a significant component of the upregulation of MUC5B mucin transcription in these structures. Distal airspace mucus accumulation could contribute to the hypoxia, pulmonary inflammation and secondary bacterial infection characteristic of severe COVID-19. These data suggest that mucolytic agents or EGFR/IL-1R antagonists may be beneficial in the clinical course of COVID-19 [77]. Additionally, dexamethasone, a recommended therapy for severely ill COVID-19 patients requiring oxygen, ventilatory support, and/or with acute respiratory distress syndrome (ARDS), have muco-inhibitory activity [78]. Dexamethasone reduced MUC5B and MUC5AC expression at both RNA and protein levels.

## 5. Other Mucolytic Drugs

The structure and mechanisms of action of Carbocysteine differ from other commonly available mucolytic drugs, such as N-acetylcysteine (NAC) and erdosteine, that exhibit free sulfhydryl (thiol) groups which cleave mucus glycoprotein bonds.

NAC, a precursor of glutathione, represents a manageable mucolytic drug in the treatment of many respiratory diseases. COPD, chronic bronchitis, and acute respiratory distress syndrome represent some of the pathological conditions in which NAC is indicated. NAC has been also evaluated as an add-on therapy in COVID-19 treatment [79]. The capability of NAC to reduce disulphides to sulphhydryl groups seems to significantly hinder the binding of SARS-CoV-2 spike protein to its molecular companion, ACE2 [80].

In addition, NAC exerts anti-inflammatory properties via the inhibition of the NLRP3 inflammasome and NFκB pathway, as well as the production of IL-6, IL-8 and TNF-α [79,80]. Its antioxidant, mucolytic action and anti-inflammatory properties support the role of NAC in COVID-19 treatment. In a recent randomized study, NAC administration (600 mg bid orally) in hospitalized COVID-19 patients, in addition to standard care, reduced the risk of severe respiratory failure (SRF) and need of mechanical ventilation, and led to a significantly lower mortality rate compared to the control group [81]. Concomitant respiratory bacterial infections among patients with SARS-CoV-2 infection has been recently reported [82]. In this respect, NAC appears to impede biofilm formation, suggesting a potential role in reducing bacteria overgrowth [83]. Another interesting drug classified in the group of the mucolytic agents but with broader pharmacological effects is erdosteine (N-(carboxymethylthioacetyl)-homocysteine thiolactone) [80]. Erdosteine (300 mg bid orally) and its metabolite Met-1 have been shown to enhance mucociliary clearance and reduce viscosity of mucoprotein solution and bacterial adhesion on epithelium [84,85,86]. In addition, Met-1 exerts anti-inflammatory activities on neutrophils and eosinophils for its scavenger activity of the O2^-^ anion that can regulate ROS production [84]. In COPD, erdosteine is able to improve the clinical score, reducing, also, the overall risk of exacerbations [87]. Erdosteine (300 mg bid orally) in addition to standard therapy in COVID-19 treatment demonstrated an improved quality of life and symptoms [88]. Perspective studies, as well as large retrospective investigations, are required to better understand the role of mucolytic agents in both early SARS-CoV-2 infections and established COVID-19 diseases.

## 6. Conclusions

The potential therapeutic benefits of mucolytics and antioxidants are attracting considerable interest for patients with SARS-CoV-2 infection. The pharmacological properties of Carbocysteine make it an ideal candidate for supportive care in COVID-19 treatment, especially in the initial stages. Increasing nasal ciliary beats within upper airways, the first site of SARS-CoV-2 infection, as well as its muco-regulatory effects, may have relevant effects on containing infection spread towards the lower airways, facilitating self-limiting outcomes and less severe disease. In addition, the extracellular scavenging of ROS radicals, suppression of cytokine storm and reduction of nucleic acid mutations may also be beneficial for COVID-19. Accordingly, the administration of Carbocysteine should be considered in both post-exposure prophylaxis and early-phase treatment of COVID-19 also in combination with other agents (monoclonal antibodies, antivirals, non-steroidal anti-inflammatory agents, inhaled corticosteroids, etc.).

## Figures and Tables

**Table 1:** Carbocysteine in respiratory diseases. AECOPD: acute exacerbation of COPD; ACE2: Angiotensin-converting enzyme 2; CBA: ciliary bend angle; CBF: ciliary beat frequency; COPD: chronic obstructive pulmonary disease; iCl^−^: intracellular Cl^−^; GC: goblet cell; HDAC2: histone deacetylase 2; HDAC3: histone deacetylase 3; ICAM-1: Intercellular adhesion molecule-1; IP-10: Interferon gamma-induced protein 10; IL: interleukin; LPS: lipopolysaccharide; MIP-1β: macrophage inflammatory proteins-1β; MUC5AC: mucin 5AC; NE: neutrophil; NF-κB: nuclear factor-κB; TGF-β1: transforming growth factor-β1; TLR4: Toll-like receptor-4; TNF-α: tumour necrosis factor alpha; ↓: reduced; ↑: increased.

Diseases	Mechanism of Action	Ref
COPD	↑ HDAC2; HDAC3 ↑ MUC5AC; ↑ GCs hyperplasia ↓ IL-8, TLR4; LPS; NEs chemotaxis	[29,30,31]
AECOPD	↓Adhesions of pathogens ↓ ICAM-1	[32]
ASTHMA	↓TGF-β1 ↓ Remodeling of the airways ↑ Cough threshold	[33,34]
COVID-19	↓ Interaction S-Protein—ACE2 ↑ CBA; CBF ↓ iCl^−^ ↓ NF-κB ↓ Hydroxyl radicals OH● ↓ IL-6, IL-8, TNF-α, IP-10, MIP-1β	[58,59,60,61,62,63,64,65]

## Data Availability

Not applicable.
